# Supplementary material for: Analysis of Biochemical and Antimicrobial Properties of Bioactive Molecules of Argemone mexicana
Source: Molecules. 2023 May 30;28(11):4428. doi: 10.3390/molecules28114428 (PMC10254925; doi:10.3390/molecules28114428)
Supplement: Supplementary file 1 [file molecules-28-04428-s001.zip › Supplementary figures.pdf]

Leaf

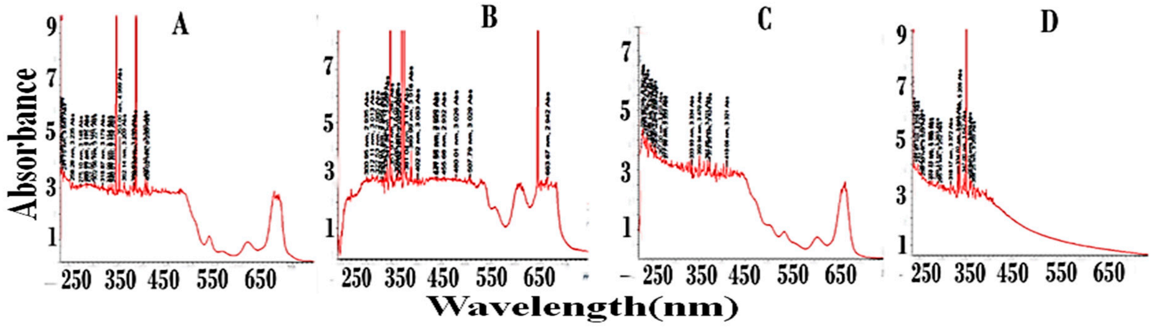

Stem

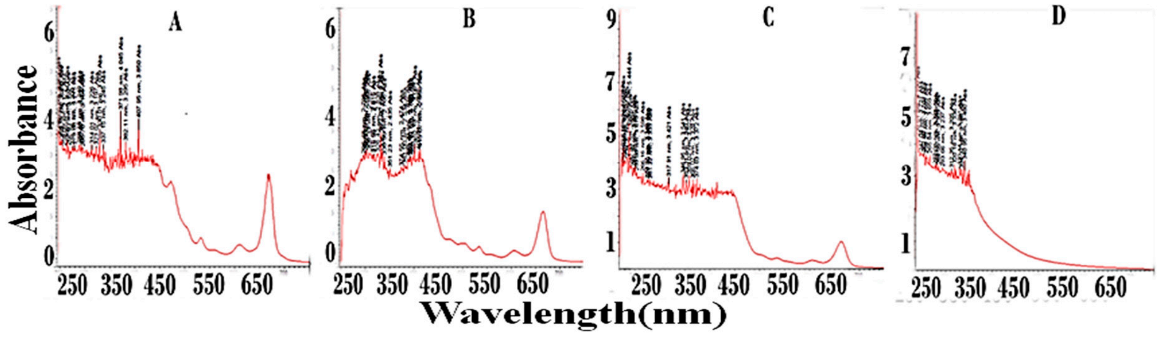

Fruit

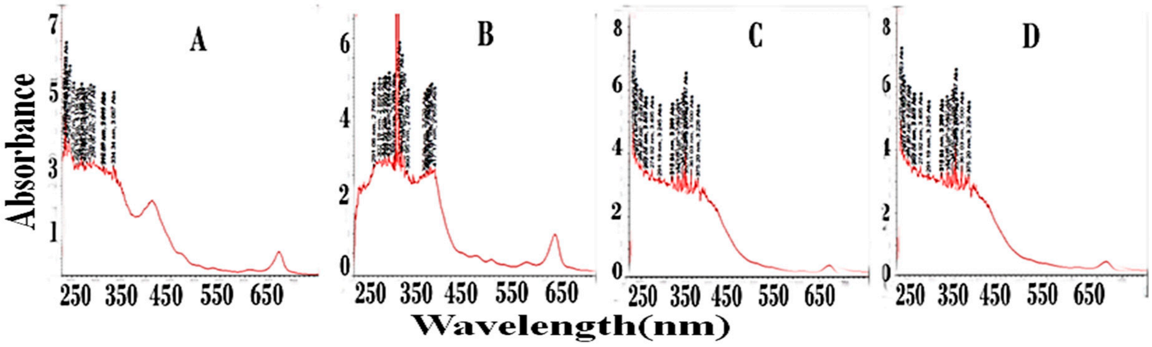

Figure S1: Spectrophotometric analysis of the phytochemicals present in various parts of*A. mexicana*, extracts prepared at room temperature (24±2^{0}C) in various solvents.A- hexane, B-ethyl acetate, C-methanol, and D-water.

Leaf

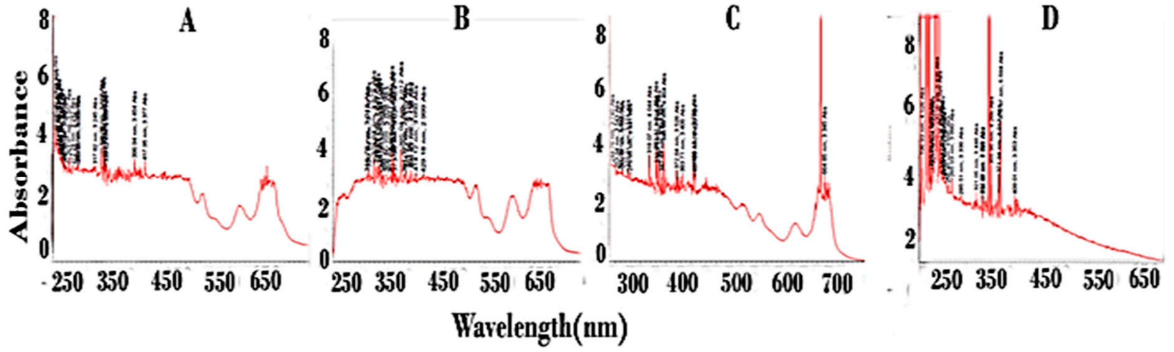

Stem

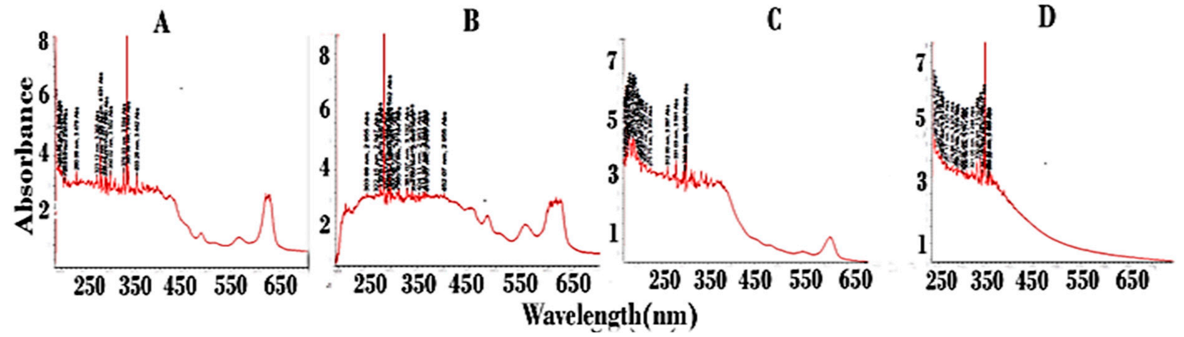

Fruit

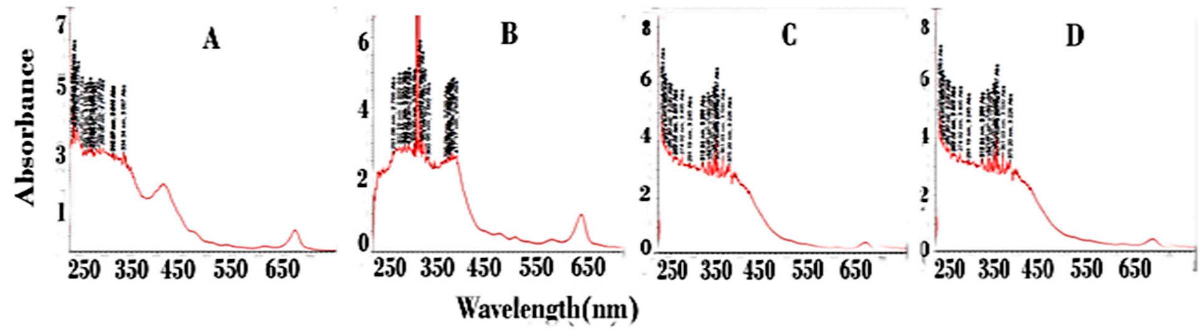

Flowers

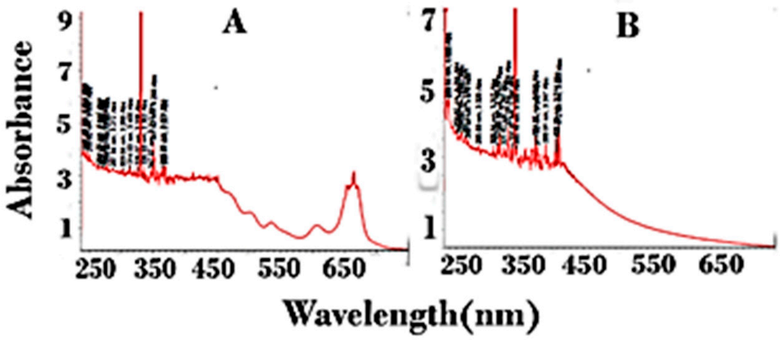

Figure S2: Spectrophotometric analysis of the phytochemicals present in various parts of*A. mexicana*, extracts prepared at high temperaturein various solvents (corresponding to their boiling points).A- hexane, B-ethyl acetate, C-methanol, and D-water.
