# Supplementary material for: Analysis of Biochemical and Antimicrobial Properties of Bioactive Molecules of Argemone mexicana
Source: Molecules. 2023 May 30;28(11):4428. doi: 10.3390/molecules28114428 (PMC10254925; doi:10.3390/molecules28114428)
Supplement: Supplementary file 1 [file molecules-28-04428-s001.zip › supplementary tables.pdf]

Table S1-Spectrophotometric analysis of *A. mexicana* extractsprepared at room temperature

Plant name	Part of plant	Solvents	Extract	No. of peaks	Wave-length	Probable phytochemical	Reference
Argemone mexicana	**Leaf**	Hexane	5 mg/ml	2	370,667	Flavonoids, Pheophytin A	[45, 46]
			250µg/ml	3	276,411,669	Flavonoids, Pheophytin A	[45, 46]
		Ethyl acetate	5 mg/ml	3	360,389,663	Flavonoids, Pheophytin A	[45, 46]
			250µg/ml	3	279,409,666	Flavonoids,	[45]
		Methanol	5 mg/ml	3	350,540,669	Flavonoids, Pheophytin A	[45, 46]
			250µg/ml	1	209	Unsaturated carbonyl compounds	-
		Aqueous	5 mg/ml	2	338,350	Flavonoids,	[45]
			250µg/ml	1	198	Unsaturated carbonyl compounds	-
	**Stem**	Hexane	5 mg/ml	2	371,667	Flavonoids, Pheophytin A	[45, 46]
			250µg/ml	3	276,410,669	Flavonoids, Pheophytin A	[45, 46]
		Ethyl acetate	5 mg/ml	3	332,402,669	Flavonoids	[1]
			250µg/ml	3	340,415,669	Flavonoids, Pheophytin A	[45, 46]
		Methanol	5 mg/ml	2	371,667	Flavonoids, Pheophytin A	[45, 46]
			250µg/ml	3	276,410,669	Flavonoids,	[45]
		Aqueous	5 mg/ml	3	332,402,669	Flavonoids,	[45]
			250µg/ml	3	340,415,669	Unsaturated carbonyl compounds	-
	**Fruit**	Hexane	5 mg/ml	3	301,410,667	Flavonoids, Pheophytin A	
			250µg/ml	2	206	Unsaturated carbonyl compounds	-
		Ethyl acetate	5 mg/ml	2	232,333	Flavonoids, Pheophytin A	[45]
			250µg/ml	1	206	Flavonoids, Pheophytin A	[45]
		Methanol	5 mg/ml	3	230,415,669	Flavonoids,	[45, 46]

						Pheophytin A	
			250µg/ml	2	232,280	Flavonoids	[45]
		Aqueous	5 mg/ml	3	332,409,666	Flavonoids, Pheophytin A	[45, 46]
			250µg/ml	1	221	Unsaturated carbonyl compounds	-

TableS2- Spectrophotometric analysis of *A. mexicana* extractsprepared at high temperature

**Plant name**	**Part of plant**	**Solvents**	**Extract**	**No. of peaks**	**Wavelength**	**Probable phytochemical**	**Reference**
A. mexicana	Leaf	Hexane	5 mg/ml	3	236, 329, 669	Flavonoids, Pheophytin A	[45, 46]
			250µg/ml	1	231	Flavonoids	[45]
		Ethyl acetate	5 mg/ml	2	385, 668	Flavonoids, Pheophytin A	[45, 46]
			250µg/ml	3	237, 410, 666	Flavonoids, Pheophytin A	[45, 46]
		Methanol	5 mg/ml	2	346 ,660	Flavonoids, Pheophytin A	[45, 46]
			250µg/ml	3	272,405,664	Flavonoids, Pheophytin A	[45, 46]
		Aqueous	5 mg/ml	2	265,376	Flavonoids,	[45]
			250µg/ml	2	205	Unsaturated carbonyl compounds	-
	Stem	Hexane	5 mg/ml	3	340,380,666	Flavonoids, Pheophytin A	[45, 46]
			250µg/ml	3	227,410,669	Flavonoids, Pheophytin A	[45, 46]
		Ethyl acetate	5 mg/ml	3	347,600,670	Flavonoids, Pheophytin A	[45, 46]
			250µg/ml	3	236,410,665	Flavonoids, Pheophytin A	[45, 46]
		Methanol	5 mg/ml	2	382, 669	Flavonoids, Pheophytin A	[45, 46]
			250µg/ml	1	206	Flavonoids,	[45]
		Aqueous	5 mg/ml	2	238, 350	Flavonoids,	[45]
			250µg/ml	1	208	Unsaturated carbonyl compounds	-
	Fruit	Hexane	5 mg/ml	3	238, 422, 669	Flavonoids, Pheophytin A	[45, 46]
			250µg/ml	1	280	Flavonoids,	[45]
		Ethyl acetate	5 mg/ml	3	343, 417, 670	Flavonoids, Pheophytin A	[45, 46]
			250µg/ml	2	236, 281	Flavonoids,	[45]
		Methanol	5 mg/ml	2	350, 669	Flavonoids, Pheophytin A	[45, 46]
			250µg/ml	2	207	Unsaturated carbonyl	-

						compounds	
		Aqueous	5 mg/ml	2	350, 142	Flavonoids,	[45]
			250µg/ml	1	206	Unsaturated carbonyl compounds	-
	Flower	Methanol	5 mg/ml	3	239, 351, 669	Flavonoids, Pheophytin A	[45, 46]
		Aqueous	250µg/ml	1	210	Unsaturated carbonyl compounds	-

The extracts of different parts of the plant, *A. mexicana*, prepared at high temperature in different solvents,
